# 1,3,7-Trimethyl-2,4-dioxo-1,2,3,4-tetra­hydro­pteridine-6-carboxylic acid hemihydrate

**DOI:** 10.1107/S1600536810007166

**Published:** 2010-02-27

**Authors:** René Faure, Nuria A. Illán-Cabeza, Sonia B. Jiménez-Pulido, Fátima M. Linares-Ordóñez, Miguel N. Moreno-Carretero

**Affiliations:** a Laboratoire de Chimie Analytique II, Université Claude Bernard, Lyon I, 69622, Villeurbanne Cedex, France; b Departamento de Química Inorgánica y Orgánica, Facultad de Ciencias Experimentales, Universidad de Jaén, Campus Universitario "Las Lagunillas" (B3), 23071 Jaén, Spain

## Abstract

In the title compound, C_10_H_10_N_4_O_4_·0.5H_2_O, the two rings of the pteridine system are nearly coplanar [dihedral angle = 4.25 (9)°]. The atoms of the carboxyl group are also coplanar with the pteridine unit [r.m.s. deviation from the mean plane of the pteridine skeleton = 0.092 (2) Å]. In the crystal, the presence of the water molecule of crystallization (O atom site symmetry 2) leads to a hydrogen-bonding pattern different  from the one shown by many carboxylic acid compounds (dimers formed through O—H⋯O hydrogen bonds between neighbouring carboxyl groups): in the present structure, the water mol­ecule, which lies on a binary axis, acts as a bridge between two mol­ecules, forming a hydrogen-bonded dimer. In addition to the hydrogen bonds, there are π–π ring stacking inter­actions involving the pyrimidine and pyrazine rings [centroid–centroid distance = 3.689 (1)Å], and two different pyrazine rings [centroid–centroid distance = 3.470 (1)Å]. Finally, there is a C—O⋯π contact involving a carboxyl­ate C—O and the pyrimidine ring with a short O⋯*Cg* distance of 2.738 (2) Å.

## Related literature

The precursor 6-acetyl-1,3,7-trimethyl­lumazine (DLMAceM) was obtained according to literature methods, see: Kim *et al.* (1999 ▶). For the structural features of both free and complexed related pteridine derivatives, see for example: Jiménez-Pulido *et al.* (2008*a* ▶,*b* ▶, 2009 ▶).
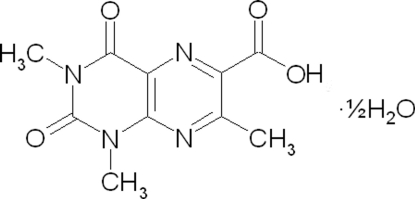


## Experimental

### 

#### Crystal data

C_10_H_10_N_4_O_4_·0.5H_2_O
*M* *_r_* = 259.23
Monoclinic, 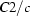

*a* = 15.7328 (19) Å
*b* = 11.5784 (16) Å
*c* = 12.4062 (18) Å
β = 106.113 (10)°
*V* = 2171.1 (5) Å^3^
*Z* = 8
Mo *K*α radiation
μ = 0.13 mm^−1^
*T* = 120 K
0.46 × 0.24 × 0.19 mm

#### Data collection

Nonius KappaCCD diffractometer
Absorption correction: multi-scan (*SADABS*; Sheldrick, 2003 ▶) *T*_min_ = 0.944, *T*_max_ = 0.976
14172 measured reflections
1970 independent reflections
1493 reflections with *I* > 2σ(*I*)
*R*_int_ = 0.038

#### Refinement

*R*[*F* ^2^ > 2σ(*F* ^2^)] = 0.055
*wR*(*F* ^2^) = 0.140
*S* = 1.21
1970 reflections
180 parameters
H atoms treated by a mixture of independent and constrained refinement
Δρ_max_ = 0.65 e Å^−3^
Δρ_min_ = −0.58 e Å^−3^

### 

Data collection: *COLLECT* (Nonius, 1998 ▶); cell refinement: *DIRAX/LSQ* (Duisenberg, 1992 ▶); data reduction: *EVALCCD* (Duisenberg *et al.*, 2003 ▶); program(s) used to solve structure: *SHELXS97* (Sheldrick, 2008 ▶); program(s) used to refine structure: *SHELXL97* (Sheldrick, 2008 ▶); molecular graphics: *Mercury* (Macrae *et al.*, 2006 ▶); software used to prepare material for publication: *WinGX* (Farrugia, 1999 ▶) and *PLATON* (Spek, 2009 ▶).

## Supplementary Material

Crystal structure: contains datablocks global, I. DOI: 10.1107/S1600536810007166/bg2332sup1.cif

Structure factors: contains datablocks I. DOI: 10.1107/S1600536810007166/bg2332Isup2.hkl

Additional supplementary materials:  crystallographic information; 3D view; checkCIF report

## Figures and Tables

**Table 1:** Hydrogen-bond geometry (Å, °)

*D*—H⋯*A*	*D*—H	H⋯*A*	*D*⋯*A*	*D*—H⋯*A*
O1*w*—H1w⋯O4^i^	0.91 (2)	1.94 (2)	2.841 (2)	172 (3)
O1*w*—H1w⋯N5^i^	0.91 (2)	2.52 (3)	2.988 (2)	113 (2)
O61—H61⋯O1*w*	0.99 (3)	1.87 (3)	2.774 (2)	151 (3)
O61—H61⋯N5	0.99 (3)	2.13 (4)	2.635 (2)	110 (2)

Symmetry code: (i) 
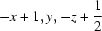
.

**Fig. 1.**
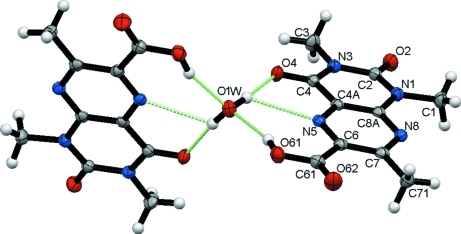
View of the H-bonds (light green broken lines) scheme for 6-carboxy-1,3,7-trimethyllumazine showing the atom labels. Thermal ellipsoids are drawn at the 50% probability level.

**Fig. 2.**
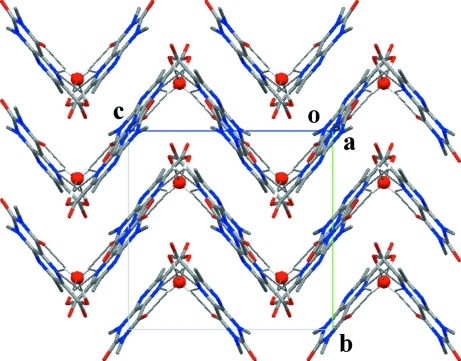
View along [100] of the molecular arrangement in the crystal.

## supplementary crystallographic information

### Comment

The interest in the 6-substituted lumazine derivatives has been increased since new coordination position pathways and new chemical and biological properties are provided while keeping the similarity to natural pterines. In this article we describe a new pteridine derivative, the 6-carboxy-1,3,7-trimetillumazine (6-carboxy-1,3,7-trimethylpteridine-2,4(1*H*,3H)-dione), which crystallizes as hemihydrate. The two rings of the pteridine system are nearly coplanar (acute dihedral angle 4.25°). The atoms of carboxylic group are also coplanar with the pteridine moiety. The presence of the water molecule makes the hydrogen bond pattern  different from the usual one in many carboxylic acid compounds: in the present structure the water molecule, which lies on a binary axis, acts like a bridge between two molecules, using its full ability for H-bond formation (Fig. 1, Table 1). In addition to the H-bonds, there are π-π ring stacking interactions which involves the pirimidine (x,y,z) and pyrazine (1/2-x, 3/2-y,-z) rings (Fig. 2). The perpendicular distances are 3.261 and 3.173 Å, the centroid-centroid separation is 3.689 Å, the dihedral angle between the planes concerned is 4.25 °. Another π-π interaction between the pyrazine ring portions at (x,y,z) and (1/2-x,3/2-y,-z) is observed. The parameters, in the same order as before mentioned, are 3.189 Å, 3.470 Å, 0.02 °, respectively, corresponding to a centroids offset of 1.368 Å. Also, there is an important C—O···π contact involving O62 and the pyrimidine ring in x, 1-y, z-1/2 with a distance between the O62 atom and the centroid of the ring of 2.738 (2) Å, a slipping angle between the O62-centroid vector and the normal to the ring of 11.4° and a C61—O62···centroid angle of 131.2 (1)° .

### Experimental

The new carboxylate ligand was prepared from the oxidation of 6-acetyl-1,3,7-trimethyllumazine with HNO_3_ (40%). This suspension was stirred at room temperature for 3 hours. The ligand was filtered off and isolated in high yield (75-80%). The pale-yellow solution was kept at room for several days, affording prismatic yellow crystals that were collected and used for X-ray diffraction studies.

(6-acetyl-1,3,7-trimethyllumazine (DLMAceM) was prepared by standard Timmis reaction between 6-amino-5-nitrosopyrimidines and 1,3-dicarbonylic derivatives by the method described by Kim *et al.*)

### Refinement

The H atoms attached to O61 and O1w were located in subsequents difference Fourier map and refined isotropically. Methyl hydrogens were fixed geometrically and treated as riding with U_iso_=1.5U_eq_(C).

### Crystal data

**Table:**

C_10_H_10_N_4_O_4_·0.5H_2_O	*F*(000) = 1080
*M**_r_* = 259.23	*D*_x_ = 1.586 Mg m^−^^3^
Monoclinic, *C*2/*c*	Mo *K*α radiation, λ = 0.71073 Å
Hall symbol: -C 2yc	Cell parameters from 1970 reflections
*a* = 15.7328 (19) Å	θ = 2.2–25.3°
*b* = 11.5784 (16) Å	µ = 0.13 mm^−^^1^
*c* = 12.4062 (18) Å	*T* = 120 K
β = 106.113 (10)°	Prism, light yellow
*V* = 2171.1 (5) Å^3^	0.46 × 0.24 × 0.19 mm
*Z* = 8	

### Data collection

**Table:**

Nonius KappaCCD diffractometer	1970 independent reflections
Radiation source: fine-focus sealed tube	1493 reflections with *I* > 2σ(*I*)
graphite	*R*_int_ = 0.038
CCD rotation images, thick slices scans	θ_max_ = 25.3°, θ_min_ = 2.2°
Absorption correction: multi-scan (*SADABS*; Sheldrick, 2003)	*h* = −18→18
*T*_min_ = 0.944, *T*_max_ = 0.976	*k* = −13→13
14172 measured reflections	*l* = −14→14

### Refinement

**Table:**

Refinement on *F*^2^	Secondary atom site location: difference Fourier map
Least-squares matrix: full	Hydrogen site location: inferred from neighbouring sites
*R*[*F*^2^ > 2σ(*F*^2^)] = 0.055	H atoms treated by a mixture of independent and constrained refinement
*wR*(*F*^2^) = 0.140	*w* = 1/[σ^2^(*F*_o_^2^) + (0.0794*P*)^2^ + 0.6244*P*] where *P* = (*F*_o_^2^ + 2*F*_c_^2^)/3
*S* = 1.21	(Δ/σ)_max_ < 0.001
1970 reflections	Δρ_max_ = 0.65 e Å^−^^3^
180 parameters	Δρ_min_ = −0.58 e Å^−^^3^
0 restraints	Extinction correction: *SHELXL97* (Sheldrick, 2008), Fc^*^=kFc[1+0.001xFc^2^λ^3^/sin(2θ)]^-1/4^
Primary atom site location: structure-invariant direct methods	Extinction coefficient: 0.0125 (14)

### Special details

**Table:**

Geometry. All esds (except the esd in the dihedral angle between two l.s. planes) are estimated using the full covariance matrix. The cell esds are taken into account individually in the estimation of esds in distances, angles and torsion angles; correlations between esds in cell parameters are only used when they are defined by crystal symmetry. An approximate (isotropic) treatment of cell esds is used for estimating esds involving l.s. planes.
Refinement. Refinement of *F*^2^ against ALL reflections. The weighted *R*-factor wR and goodness of fit *S* are based on *F*^2^, conventional *R*-factors *R* are based on *F*, with *F* set to zero for negative *F*^2^. The threshold expression of *F*^2^ > σ(*F*^2^) is used only for calculating *R*-factors(gt) etc. and is not relevant to the choice of reflections for refinement. *R*-factors based on *F*^2^ are statistically about twice as large as those based on *F*, and *R*- factors based on ALL data will be even larger.

### Fractional atomic coordinates and isotropic or equivalent isotropic displacement parameters (Å^2^)

**Table:**

	*x*	*y*	*z*	*U*_iso_*/*U*_eq_	
O1w	0.5000	0.75980 (19)	0.2500	0.0271 (5)	
N1	0.16439 (11)	0.99057 (14)	−0.04043 (14)	0.0213 (4)	
C2	0.22500 (13)	1.06607 (18)	−0.06308 (17)	0.0217 (5)	
N3	0.31401 (11)	1.03800 (14)	−0.02157 (14)	0.0215 (4)	
C4	0.34674 (13)	0.94797 (17)	0.04960 (16)	0.0201 (5)	
C4A	0.27938 (13)	0.87743 (17)	0.07728 (15)	0.0181 (5)	
N5	0.30569 (11)	0.79181 (13)	0.14868 (13)	0.0190 (4)	
C6	0.24465 (13)	0.73144 (16)	0.17889 (16)	0.0201 (5)	
C7	0.15435 (14)	0.75701 (17)	0.13777 (16)	0.0211 (5)	
N8	0.12794 (11)	0.84253 (14)	0.06406 (13)	0.0209 (4)	
C8A	0.18987 (13)	0.90176 (17)	0.03424 (15)	0.0189 (5)	
C1	0.07098 (14)	1.0169 (2)	−0.09039 (18)	0.0294 (6)	
O2	0.20238 (10)	1.15152 (13)	−0.11888 (12)	0.0303 (4)	
C3	0.37550 (14)	1.11665 (19)	−0.05308 (19)	0.0289 (5)	
O4	0.42558 (9)	0.92948 (12)	0.08549 (12)	0.0257 (4)	
C61	0.28018 (14)	0.63747 (18)	0.26029 (17)	0.0246 (5)	
O61	0.36742 (10)	0.63064 (13)	0.30013 (12)	0.0283 (4)	
O62	0.23356 (10)	0.56896 (13)	0.28942 (13)	0.0362 (5)	
C71	0.08347 (14)	0.69621 (18)	0.17313 (18)	0.0267 (5)	
H1W	0.5284 (19)	0.809 (2)	0.305 (2)	0.065 (10)*	
H1A	0.0366	0.9451	−0.1014	0.044*	
H1B	0.0636	1.0550	−0.1630	0.044*	
H1C	0.0501	1.0683	−0.0404	0.044*	
H3A	0.4363	1.0901	−0.0197	0.043*	
H3B	0.3684	1.1945	−0.0257	0.043*	
H3C	0.3631	1.1182	−0.1350	0.043*	
H61	0.398 (2)	0.694 (3)	0.273 (3)	0.068 (9)*	
H71A	0.0264	0.7330	0.1379	0.040*	
H71B	0.0958	0.7004	0.2549	0.040*	
H71C	0.0814	0.6151	0.1499	0.040*	

### Atomic displacement parameters (Å^2^)

**Table:**

	*U*^11^	*U*^22^	*U*^33^	*U*^12^	*U*^13^	*U*^23^
O1w	0.0226 (11)	0.0273 (12)	0.0279 (12)	0.000	0.0013 (9)	0.000
N1	0.0182 (9)	0.0239 (9)	0.0210 (9)	0.0019 (7)	0.0040 (7)	0.0038 (7)
C2	0.0226 (11)	0.0241 (11)	0.0182 (10)	0.0020 (9)	0.0055 (8)	−0.0001 (9)
N3	0.0204 (9)	0.0235 (9)	0.0206 (9)	−0.0005 (7)	0.0059 (7)	0.0031 (7)
C4	0.0211 (11)	0.0217 (11)	0.0167 (10)	0.0000 (9)	0.0036 (8)	−0.0015 (8)
C4A	0.0205 (11)	0.0188 (10)	0.0153 (10)	0.0026 (8)	0.0056 (8)	−0.0013 (8)
N5	0.0212 (9)	0.0189 (9)	0.0168 (8)	0.0006 (7)	0.0053 (7)	−0.0021 (7)
C6	0.0230 (11)	0.0201 (11)	0.0181 (10)	−0.0025 (9)	0.0073 (8)	−0.0029 (8)
C7	0.0251 (11)	0.0213 (11)	0.0178 (10)	−0.0019 (9)	0.0073 (9)	−0.0048 (8)
N8	0.0198 (9)	0.0228 (9)	0.0208 (9)	−0.0002 (7)	0.0067 (7)	−0.0017 (7)
C8A	0.0216 (11)	0.0199 (11)	0.0152 (10)	0.0009 (8)	0.0053 (8)	−0.0040 (8)
C1	0.0193 (11)	0.0373 (13)	0.0296 (12)	0.0047 (10)	0.0037 (9)	0.0078 (10)
O2	0.0283 (9)	0.0298 (9)	0.0318 (9)	0.0052 (7)	0.0065 (7)	0.0114 (7)
C3	0.0242 (12)	0.0299 (12)	0.0332 (12)	−0.0037 (10)	0.0091 (10)	0.0082 (10)
O4	0.0168 (8)	0.0304 (8)	0.0288 (8)	0.0000 (6)	0.0047 (6)	0.0050 (6)
C61	0.0276 (12)	0.0251 (12)	0.0209 (11)	−0.0005 (9)	0.0064 (9)	0.0003 (9)
O61	0.0265 (8)	0.0282 (9)	0.0277 (8)	0.0026 (7)	0.0036 (7)	0.0056 (7)
O62	0.0352 (9)	0.0340 (9)	0.0391 (10)	−0.0040 (8)	0.0100 (8)	0.0148 (8)
C71	0.0234 (11)	0.0314 (12)	0.0265 (12)	−0.0048 (10)	0.0093 (9)	−0.0009 (9)

### Geometric parameters (Å, °)

**Table:**

O1w—H1W	0.90 (3)	N1—C2	1.379 (3)
O4—C4	1.215 (2)	N1—C1	1.460 (3)
C4A—N5	1.317 (3)	O61—C61	1.326 (3)
C4A—C8A	1.389 (3)	O61—H61	0.98 (3)
C4A—C4	1.453 (3)	O62—C61	1.202 (3)
N8—C8A	1.325 (3)	C7—C71	1.484 (3)
N8—C7	1.334 (3)	C71—H71A	0.9800
N5—C6	1.323 (3)	C71—H71B	0.9800
N3—C4	1.371 (3)	C71—H71C	0.9800
N3—C2	1.390 (3)	C3—H3A	0.9800
N3—C3	1.459 (3)	C3—H3B	0.9800
O2—C2	1.203 (2)	C3—H3C	0.9800
C8A—N1	1.367 (3)	C1—H1A	0.9800
C6—C7	1.401 (3)	C1—H1B	0.9800
C6—C61	1.484 (3)	C1—H1C	0.9800
			
N5—C4A—C8A	120.46 (18)	O2—C2—N1	121.85 (19)
N5—C4A—C4	117.93 (18)	O2—C2—N3	120.80 (18)
C8A—C4A—C4	121.56 (18)	N1—C2—N3	117.32 (17)
C8A—N8—C7	117.54 (18)	C7—C71—H71A	109.5
C4A—N5—C6	118.07 (18)	C7—C71—H71B	109.5
C4—N3—C2	125.18 (17)	H71A—C71—H71B	109.5
C4—N3—C3	119.27 (17)	C7—C71—H71C	109.5
C2—N3—C3	115.45 (16)	H71A—C71—H71C	109.5
N8—C8A—N1	118.56 (18)	H71B—C71—H71C	109.5
N8—C8A—C4A	122.21 (18)	N3—C3—H3A	109.5
N1—C8A—C4A	119.22 (18)	N3—C3—H3B	109.5
N5—C6—C7	121.83 (18)	H3A—C3—H3B	109.5
N5—C6—C61	114.46 (18)	N3—C3—H3C	109.5
C7—C6—C61	123.70 (18)	H3A—C3—H3C	109.5
C8A—N1—C2	121.70 (17)	H3B—C3—H3C	109.5
C8A—N1—C1	121.06 (17)	O62—C61—O61	120.23 (19)
C2—N1—C1	116.92 (17)	O62—C61—C6	122.8 (2)
C61—O61—H61	112.3 (17)	O61—C61—C6	116.92 (18)
O4—C4—N3	122.20 (18)	N1—C1—H1A	109.5
O4—C4—C4A	123.50 (18)	N1—C1—H1B	109.5
N3—C4—C4A	114.30 (17)	H1A—C1—H1B	109.5
N8—C7—C6	119.86 (18)	N1—C1—H1C	109.5
N8—C7—C71	115.99 (19)	H1A—C1—H1C	109.5
C6—C7—C71	124.13 (18)	H1B—C1—H1C	109.5

### Hydrogen-bond geometry (Å, °)

**Table:**

*D*—H···*A*	*D*—H	H···*A*	*D*···*A*	*D*—H···*A*
O1w—H1w···O4^i^	0.91 (2)	1.94 (2)	2.841 (2)	172 (3)
O1w—H1w···N5^i^	0.91 (2)	2.52 (3)	2.988 (2)	113 (2)
O61—H61···O1w	0.99 (3)	1.87 (3)	2.774 (2)	151 (3)
O61—H61···N5	0.99 (3)	2.13 (4)	2.635 (2)	110 (2)

Symmetry codes: (i) −*x*+1, *y*, −*z*+1/2.
